# Planning ahead: Development of a brief, self‐report measure to assess advance care planning self‐efficacy and readiness in informal caregivers of persons with cognitive decline

**DOI:** 10.1002/alz70858_106915

**Published:** 2025-12-26

**Authors:** Ryan C Thompson White, Virginia T Gallagher, Shannon E Reilly, M. Agustina Rossetti, Elizabeth Boyd, Samantha Fields, Jill Guiffre, Jessica Samet, Carol A Manning

**Affiliations:** ^1^ University of Virginia, Charlottesville, VA, USA

## Abstract

**Background:**

Advance care planning (ACP) is a process that helps individuals identify and communicate their preferences regarding end‐of‐life decisions and future medical needs. It is an ongoing and evolving process, particularly for informal caregivers of persons with cognitive decline (PWCD), whose care recipient's (CR) ability to decide and express their wishes may change as their disease progresses. Informal PWCD caregivers may not know their CR's preference for end‐of‐life care or feel prepared to follow through on established preferences on their CR's behalf. While several formal assessments of ACP have been previously published, many are lengthy or not psychometrically validated to assess ACP self‐efficacy and preparedness in informal PWCD caregivers who are actively involved in ACP with their CR; therefore, the present study aims to address this current gap in the literature.

**Method:**

The present study utilized a conceptual framework based on Behavior Change Theory, positing that knowledge, self‐efficacy, and preparedness are required for ACP to be translated into action (Sudore et al., 2013). A team of nine healthcare providers involved in the provision of clinical care in a Memory Disorders Clinic identified existing self‐report ACP measures with the goal of developing a brief, self‐report measure that would adequately assess ACP self‐efficacy and preparedness in informal PWCD caregivers who are engaging in ACP actions for their CR.

**Result:**

After reviewing PubMed, Google Scholar, Medline, and PsycINFO, 10 publications describing formal measures of ACP were identified. These publications were reviewed in a focus group of four clinical neuropsychologists, one neuropsychology fellow, and four dementia care coordinators, with the goal of creating a brief, self‐report measure to assess ACP self‐efficacy and readiness in informal PWCD caregivers. Keywords used during the literature review, item selection criteria, and an overview of the proposed survey items will be presented.

**Conclusion:**

The proposed ACP survey may be useful to clinicians and researchers evaluating ACP self‐efficacy and preparedness in informal PWCD caregivers who are engaging in ACP activities with their CR. Future directions include administering the proposed survey to a clinical sample of informal PWCD caregivers to evaluate its psychometric properties, including internal consistency, test‐retest reliability, and construct validity.

